# Lipid metabolism contribute to the pathogenesis of IgA Vasculitis

**DOI:** 10.1186/s13000-021-01185-1

**Published:** 2022-02-11

**Authors:** Ying Liu, Min Wen, Qingnan He, Xiqiang Dang, Shipin Feng, Taohua Liu, Xuewei Ding, Xiaoyan Li, Xiaojie He

**Affiliations:** 1 Department of Pediatrics, The Third Xiangya Hospital, Central South University, Changsha, Hunan China; 2 Institute of Pediatrics, The Second Xiangya Hospital, Central South University, Changsha, China; 3 Laboratory of Pediatric Nephrology, Institute of Pediatrics, The Second Xiangya Hospital, Central South University, Changsha, China; 4 Department of Pediatric Nephrology, Chengdu Women’s and Children’s Central Hospital, School of Medicine, University of Electronic Science and Technology of China, Chengdu, Sichuan China

**Keywords:** IgA vasculitis, IgA vasculitis with nephritis, Lipid metabolism, Serum lipidome

## Abstract

**Background and objectives:**

The underlying mechanism of IgA vasculitis (IgAV) and IgA vasculitis with nephritis (IgAVN) remains unclear. Therefore, there are no accurate diagnostic methods. Lipid metabolism is related to many immune related diseases, so this study set out to explore the relationship of lipids and IgAV and IgAVN.

**Methods:**

Fifty-eighth patients with IgAV and 28 healthy controls were recruited, which were divided into six separate pools to investigate the alterations of serum lipids according to the clinical characteristics: healthy controls group (HCs) and IgAV group (IgAVs), IgAVN group (IgAV-N) and IgAV without nephritis group (IgAV-C), initial IgAV group (IgAV0) and IgAV in treatment with glucocorticoids group (IgAV1).

**Results:**

31 identified lipid ions significantly changed in IgAVs with *p* < 0.05, variable importance of the projection (VIP) > 1 and fold change (FC) > 1.5. All these 31 lipid ions belong to 6 classes: triacylglycerols (TG), phosphatidylethanolamine (PE), phosphatidylcholine (PC), phosphatidylserine, ceramide, and lysophosphatidylcholine. TG (16:0/18:1/22:6) +NH4 over 888875609.05, PC (32:1) +H over 905307459.90 and PE (21:4)-H less than 32236196.59 increased the risk of IgAV significantly (OR>1). PC (38:6) +H was significantly decreased (*p* < 0.05, VIP>1 and FC>1.5) in IgAVN. PC (38:6) less than 4469726623 conferred greater risks of IgAV (OR=45.833, 95%CI: 6.689~341.070).

**Conclusion:**

We suggest that lipid metabolism may affect the pathogenesis of IgAV via cardiovascular disease, insulin resistance, cell apoptosis, and inflammation. The increase of TG(16:0/18:1/22:6) + NH4, and PC(32:1) + H as well as PE (21:4)-H allow a good prediction of IgAV. PE-to-PC conversion may participate in the damage of kidney in IgAV. PC (38:6) + H may be a potential biomarker for IgAVN.

**Supplementary Information:**

The online version contains supplementary material available at 10.1186/s13000-021-01185-1.

## Introduction

IgA vasculitis (IgAV), also referred to as Henoch-Schönlein purpura (HSP), is the most common systemic vasculitis in children with an annual incidence of 3 ~ 26.7 cases per 100,000 [1]. IgAV is an autoimmune disease characterized by IgA depositing on arterioles, capillaries, and venules. Skin, gastrointestinal tract, joints and kidneys are the main involved organs [2]. As a result, purpura, arthralgia, and abdominal are the “classic triad” of IgAV. IgAV was first reported by Heberden in 1802, however, the cause of it still remains unclear during the past two hundred years [3, 4]. At present, most researchers believe IgAV is related to the high level of IgA1-AECA, which binds to small vessels to induce cytokines that recruit neutrophils activated by the interaction between IgA1 and its receptor FcαRI, resulting in inflammation [3]. Nevertheless, there is no specific diagnostic laboratory test available as markers of IgAV up to date [7, 8]. When severe symptoms occur, glucocorticoids are the main choice for IgAV. IgA vasculitis with nephritis (IgAVN) is an important factor in the poor prognosis of IgAV and about 30% of IgAV patients suffer from nephritis9. Most methods for diagnosis and treatment of IgAVN are based on studies of IgA nephropathy, which is also closely related with galactose-deficient IgA1 [10, 11]. So, the mechanism of IgAV and IgAVN required further investigation to define potential biomarkers to assist in the early detection and accurate diagnosis for improving the prognosis of IgAV.

Lipidomics, a sub-discipline of metabolomics, has become a hot spot in scientific research. Lipids play significant roles in human health as important components of biological membranes, the major source of energy, signaling molecules, and second messengers [12]. The Lipid Metabolites and Pathways Strategy Consortium (LIPID MAPS) has classified lipids into eight categories: fatty acyls (FA), glycerolipids (GL), glycerophospholipids (GP), sphingolipids (SP), sterol lipids (ST), prenol lipids (PR), saccharolipids (SL), and polyketides (PK) [13]. Fatty acid is one of the most common lipid class and it can be divided to saturated fatty acids (e.g. palmitic acid), monounsaturated fatty acids (e.g. oleic acid) and polyunsaturated fatty acids (e.g. docosahexaenoic acid or linoleic acid), according to chain length and degree of saturation. Among these, saturated fatty acids were reported to be related with inflammation with insulin-resistant states and atherosclerosis, whereas oleic acid and polyunsaturated omega-3 fatty acids are anti-inflammatory and protective against these metabolic diseases [14]. GL are esterified by glycerol and fatty acids, according to the number of which, GL can be classified to be monoacylglycerols (MG), diacylglycerols (DG) and triacylglycerols (TG). Higher levels of DG and TG increase the risk of type 2 diabetes [15]. GP consist of DG and ether glycerophospholipids. With respect to the different phosphate groups, GP were categorized into different lipid classes, such as phosphatidylglycerol (PG), phosphatidylcholine (PC), phosphatidylethanolamine (PE). SP were associated with obesity, insulin resistance, muscle function, β-cell exhaustion, the inflammatory response, vascular complications, and cardiac failure [16–19]. Ceramide (Cer) and sphingomyelin were the most common SP. ST included sterols, steroids, secosteroids, bile acids with derivatives, steroid conjugates and so on. Cholesterol, a kind of sterols, is an important component of the cellular membrane in animals. Vitamin E, Vitamin K and Coenzyme Q10 were well known PR. SL were fatty acids with the sugar moiety. Many *polyketides* are clinically important, with antimicrobial, anticancer and immunosuppressive properties [20, 21].

Lipidomics has been established to play an important role in many diseases, such as IgA nephropathy, systemic lupus erythematosus, major depressive disorder, type 2 diabetes [15, 22–25]. In IgAV, paraoxonase1 (PON1), a high density lipoprotein (HDL)-associated enzyme preventing lipid peroxidation was confirmed to be associate with the development and progression of arterial damage. Meanwhile, the differentially expressed proteins in IgAVN were reported to be enriched in lipid metabolism [26].

In this study, we sought to discover the serum lipid alternations in IgAV and IgAVN children by using Liquid Chromatography with tandem mass spectrometry (LCMS/MS) [27], trying to find some biomarkers in IgAV and IgAVN disease.

## Methods

### Ethical statement

A total of 86 individuals under 18 years old participated in our research, and informed consent was signed by their guardians. The experimental protocol was reviewed and approved by the Institutional Review Board of The Second Xiangya Hospital of Central South University (XXJ2018–11).

### Participants and grouping

A total of 86 individuals under 18 years old participated in our research, and informed consent was signed by their guardians. All participants were recruited from The Second Xiangya Hospital of Central South University between November 2018 and May 2019(Hunan Changsha, China). 58 IgAV patients were diagnosed based on EULAR/PRINTO/PRES 2008 criteria for Henoch-Schonlein purpura: Purpura or petechiae (mandatory) with lower limb predominance [7]. The healthy control group (HCs group) consisted of 28 healthy children without any disease or drug-using histories in recent 6 months, confirming by screening questionnaires. All the participants with previous chronic inflammatory diseases, chronic kidney disease, other systemic vasculitis or immunological diseases were excluded.

In IgAVs groups, according to the renal damage and glucocorticoids treatment, they were divided into four groups. The diagnosis of IgAV with nephritis (IgAV-N group) was made when patients had biopsy specimen–proven renal involvement with mesangial IgA in electron microscopy. IgAV patients without nephritis (IgAV-C group) were consistent with IgAV patients without proteinuria and hematuria on urinalysis. Initial IgAV patients (IgAV0 group) means those in the early onset did not receive any treatments, and IgAV1 groups were defined as IgAV patients with glucocorticoids treatment with or without other treatment measures.

### Sample collection and preparation

Blood samples were collected from vein in the morning after an overnight fasting (> 8 h). The quality control (QC) sample was prepared from 10 μL of each test sample. The fasting blood was collected in 5 mL vacutainer tubes containing heparin lithium, then the samples were centrifuged for 15 min (1500×g, 4 °C). Each aliquot (100 μL) of the plasma sample was stored at − 80 °C until the ultra-high-performance liquid chromatography equipped with Liquid Chromatography with tandem mass spectrometry (LC-MS/MS) analysis. The plasma samples were thawed at 4 °C and 100 μL aliquots were mixed with 200 μL water and then added to 240 μL of cold methanol/acetonitrile, again mixed well. After that, 800 μL MTBE were added and it was mixed well. The homogenate solution was followed by sonication in water for about 20 min and placed at room temperature for another 30 min. After centrifugation for 15 min (1400×g, 10 °C), the upper organic phase was collected and dried with nitrogen gas (Changsha Ruichong Gas Co., LTD). Before LC-MS/MS, each sample was added to 200 μL isopropanol:acetonitrile (90:10, v/v)**,** vortexed and centrifuged for 15 min (1400×g, 10 °C) and the supernatant was instantaneously injected into the chromatograph.

### LC-MS/MS analysis

These samples were performed on an ultra-performance liquid chromatography (UPLC) Nexera LC-30A (Agilent Technologies, Santa-Clara, California, USA). The injection volume was 3 μl, flow rate was 300 ml/ min and the column temperature was 45 °C. The mobile phase comprised of A, 10 mM ammonium formate acetonitrile solution (acetonitrile:water = 6:4, v/v) and B, 10 mM ammonium formate acetonitrile isopropyl alcohol solution (acetonitrile:isopropanol = 1:9, v/v). The program of gradient elution included 30% (B) at 0–2 min, a linear gradient from 30 to 100% (B) at 2-25 min, and 30% (B) at 25-35 min. During the analysis process, we used an automatic sample injector (10 °C) and continuous sample analysis using random order was performed to correct the impact of instrument signal fluctuation.

After the UHPLC separation, mass spectrometry was performed using a Q-Exactive plus mass spectrometer (Thermo Scientific™).

Electrospray ionization (ESI) positive ion and negative ion modes were used respectively, under the following condition:

- Positive: Heater Temp 300 °C, Sheath Gas Flow rate 45 arb, Aux Gas Flow Rate15 arb, Sweep Gas Flow Rate 1arb, spray voltage 3.0KV, Capillary Temp 350 °C, S-Lens RF Level 50%. MS1 scan ranges: 200–1800.
- Negative: Heater Temp 300 °C, Sheath Gas Flow rate 45arb, Aux Gas Flow Rate 15arb, Sweep Gas Flow Rate 1arb, spray voltage 2.5KV, Capillary Temp 350 °C, S-Lens RF Level 60%. MS1 scan ranges: 250–1800

According to the methods used which were each fully scanned (full scan), 10 pieces of map (MS2 scan, HCD) were collected, and the lipid molecules and quality of the lipid debris charge ratio were collected.

The resolution of MS2 at M/Z200 was 17,500 AND The resolution of MS2 at M/Z200 was 17,500,

### Lipidomics data analysis

LipidSearch software version 4.1 (Thermo Scientific™) was used for data processing, such as peak identification, lipid identification, peak extraction, peak integration, and peak alignment. Lipid molecules with a RSD > 30% were removed.

The missing value in the group with > 50% of lipid molecules was also deleted and the abundance values were obtained using total peak area normalization based on LipidSearch data extraction. The application software SIMCA-P 14.1 (Umetrics, Umea, Sweden) was used for pattern recognition. *p* value< 0.05 and VIP > 1 was considered statistically significant. Fold change (FC) *> 1.5* was considered large differences.

### Statistical analysis

In order to access the relationship among changed lipidions and the severity of IgAV or IgAVN, optimal curve correlation of lipidions and multiple regression analysis of all the changed lipidions and IgAV or IgAVN were performed using SPSS (26.0). A value of *p* < 0.05 was considered statistically significant.

## Results

### The clinical and demographic characteristics of all the subjects

Twenty-eight healthy controls (HCs) (male/female: 14/14) and 58 IgAV patients (IgAVs) (male/female: 28/30) were included in this research. There were no significant differences in sex and age between the two groups. Body mass index (BMI) of IgAV patients were statistically higher (*p* = 0.01). In these 58 IgAV children, 15 (25.86%) children were initially diagnosed without any treatments (IgAV0), 30 (51.72%) were in treatment with glucocorticoids (IgAV1), and 13 patients had stopped medication after drug treatment (22.41%), purpura (48.28%), arthralgia (20.69%), and abdominal symptoms (20.69%) were the main clinical features of these patients. 13 (22.41%) IgAVs were diagnosed with IgAVN (IgAV-N) from electron microscopy (patients detected only by light microscopy were excluded). 28 (48.28%) IgAVs were defined as IgAV without nephritis (IgAV-C) by the absence of proteinuria and hematuria on urinalysis. The clinical and demographic characteristics of the subjects are presented in Table 1.

**Table 1:** The clinical and demographic characteristics of all the subjects

Characteristics of different groups	IgAVs	HCs	IgAV-N	IgAV-C	IgAV0	IgAV1	***p*** value		
							IgAVs vs HCs	IgAV-N vs IgAV-C	IgAV0 vs IgAV1
Patients (*n*)	58	28	13	28	15	30	NA	NA	NA
Male (*n*, %) ^a^	28 (48.3%)	14 (50.0%)	7 (50.0%)	16 (53.6%)	7 (46.7%)	16 (53.3%)	0.881	0.82	0.881
Age (years)									
	2~14	3~17	4~14	7~14	2~14	4~14	NA	NA	NA
Mean ± SD ^b^	9.17 ± 3.08	10.61 ± 3.67	10.23 ± 2.52	8.50 ± 2.90	8.27 ± 3.67	9.80 ± 2.76	0.061	0.072	0.12
BMI (kg/m^2^)									
Mean ± SD ^b^	16.47 ± 2.85	18.57 ± 4.51	17.14 ± 1.32	15.88 ± 1.73	15.96 ± 2.10	16.83 ± 3.46	0.01	0.153	0.38
Symptoms									
Purpura	28 (48.28%)	0(0.00%)	2(15.38%)	14(50.00%)	15(100.00%)	14(46.67%)	NA	NA	NA
Arthralgia	12 (20.69%)	0(0.00%)	1(7.69%)	6(21.43%)	6(40.00%)	6(20.00%)	NA	NA	NA
Abdominal pain	12 (20.69)	0(0.00%)	0(0.00%)	8(28.57%)	6(40.00%)	6(20.00%)	NA	NA	NA
Renal biopsy	13 (22.41%)	0(0.00%)	13(100.00%)	0(0.00%)	1(6.67%)	14(46.67%)	NA	NA	NA
Haematuria	21 (36.21%)	0(0.00%)	2(15.38%)	0(0.00%)	2(13.33%)	6(20.00%)	NA	NA	NA
Proteinuria	13 (22.41%)	0(0.00%)	8(61.84%)	0(0.00%)	2(13.33%)	13(43.33%)	NA	NA	NA

^a^Analyzed by the Chi-square test

^b^Analyzed by the ANOVA followed by post hoc comparison of the groups using the Bonferroni test

NA means it cannot be calculated

### Lipidomics analysis of plasma obtained from IgAV subjects and HCs

**Fig. 1:**
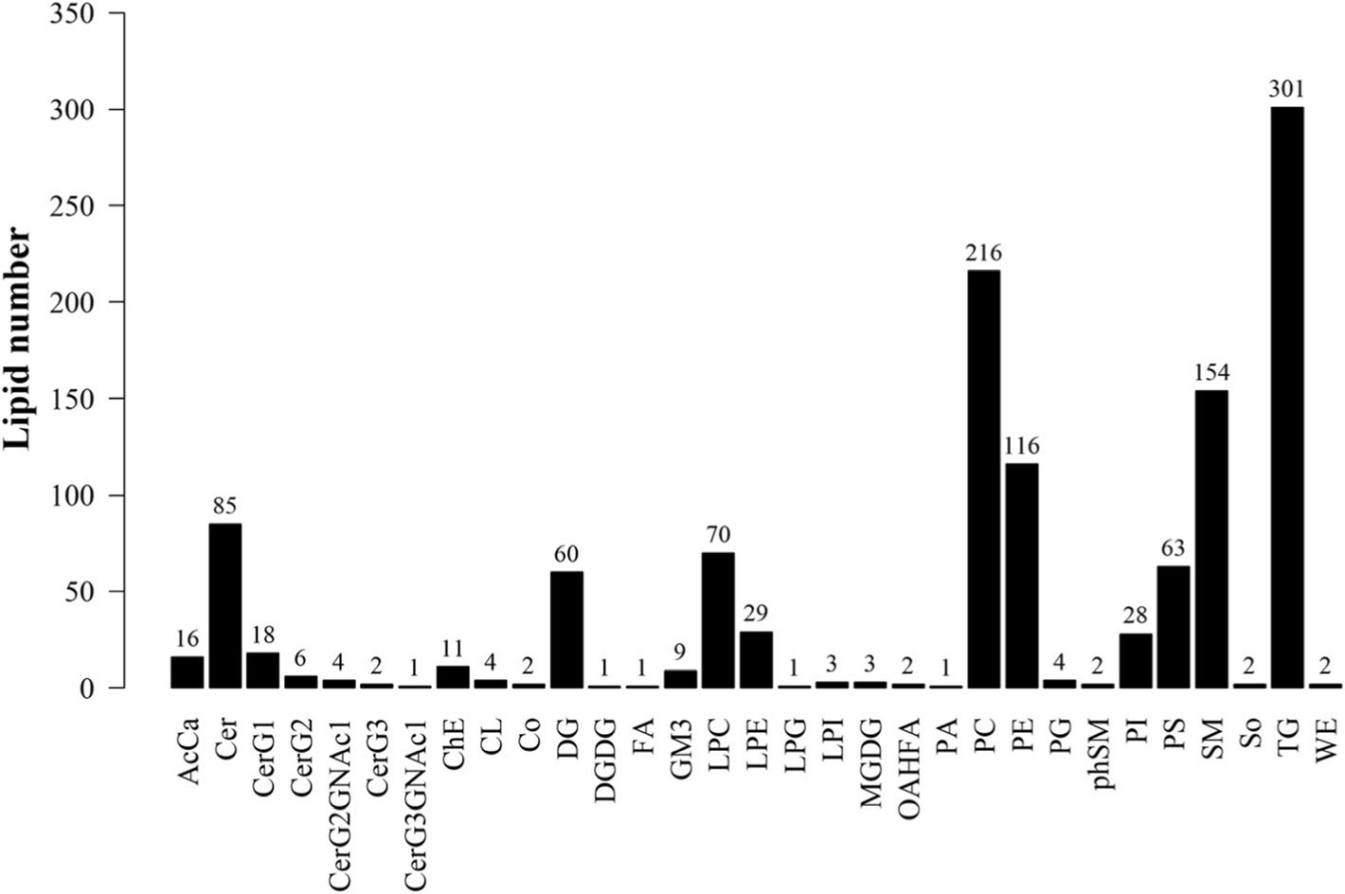
all the lipids detected in plasma samples in HCs and IgAVs. All the 1217 species of lipids detected belonged to 31 classes, of which TG (301), PC (216), SM (154) and PE (116) were the predominant lipid

The data obtained from negative and positive ion modes on the UHPLC-Orbitrap MS were analyzed to qualitatively identify then perform quantification. A total of 31 classes and 1217 species of lipids (including homomers) were analyzed using the Lipid Search software version 4.1. Among these 31 lipid classes, classes possessing the highest number of lipid species were TG (301), PC (216), SM (154) and PE (116) **(**Fig. 1**)**.

In order to identify the differences of lipid classes in IgAVs, Wilcoxon test was performed and a single lipid class, GM3, was significantly associated (*p* = 0.000454) with IgAV. However, the expression changes of all these significant lipid classes (*p* < 0.05) were not obvious (FC < 1.5). To validate the reproducibility of the results, comparison was made among the basic peak chromatogram (BPC) of QC samples from both negative and positive ion modes **(**Fig. S1**)**. The results showed an overlap between response strength and retention time of the chromatographic peak; the experimental repeatability was also deemed good. To look for differences of lipidions between IgAVs and HCs, OPLS-DA score plot and permutation test results were displayed **(**Fig. S2**)**. There were significant differences between these two groups. We performed a preliminary screen for differences under multivariate statistical analyses (VIP > 1) based on the OPLS-DA model, and we also identified the significance under univariate statistical analyses (*P* < 0.05). Finally, we screened 31 lipidions with multiple change greater than 1.5.

**Fig. 2:**
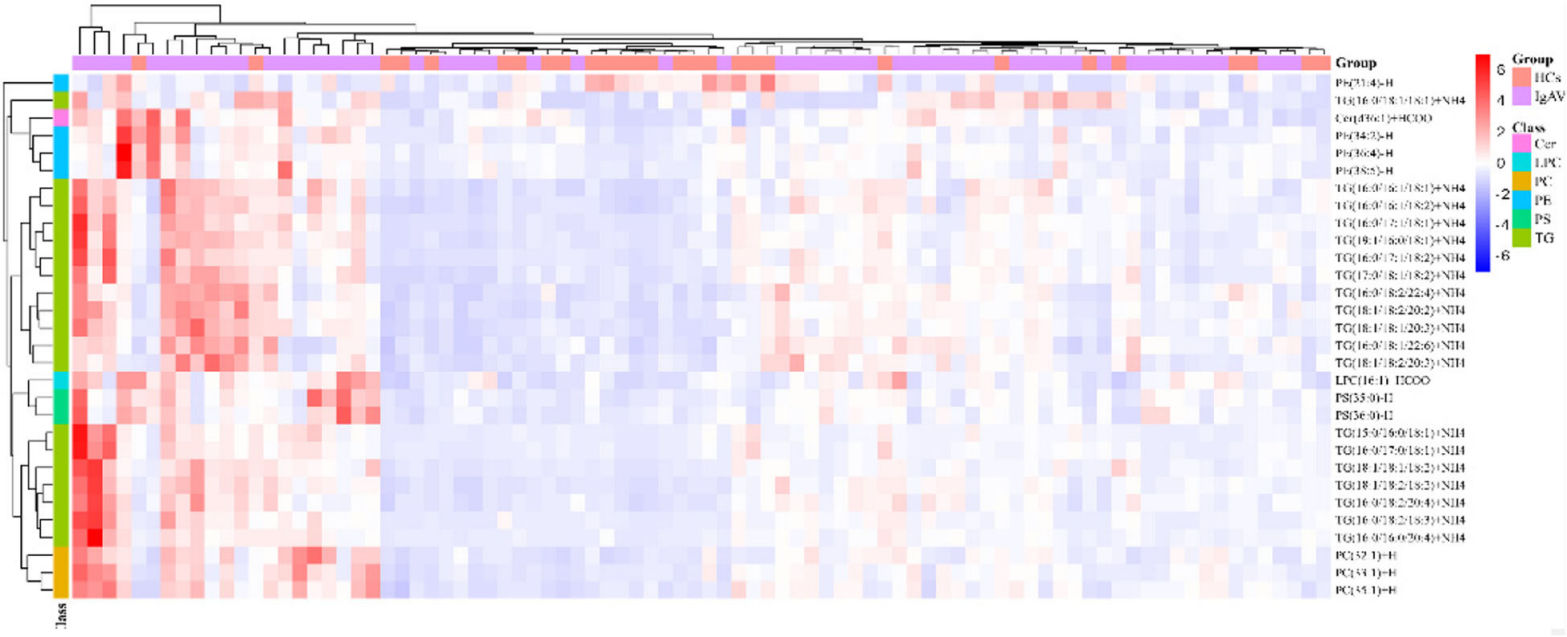
The heat plot for the differential lipids in IgAVs vs HCs. Decreased lipidions colored blue and increased lipidions colored red. All Most of these altered lipidions rose in IgAV patients, except for PE (21:4)-H

**Fig. 3:**
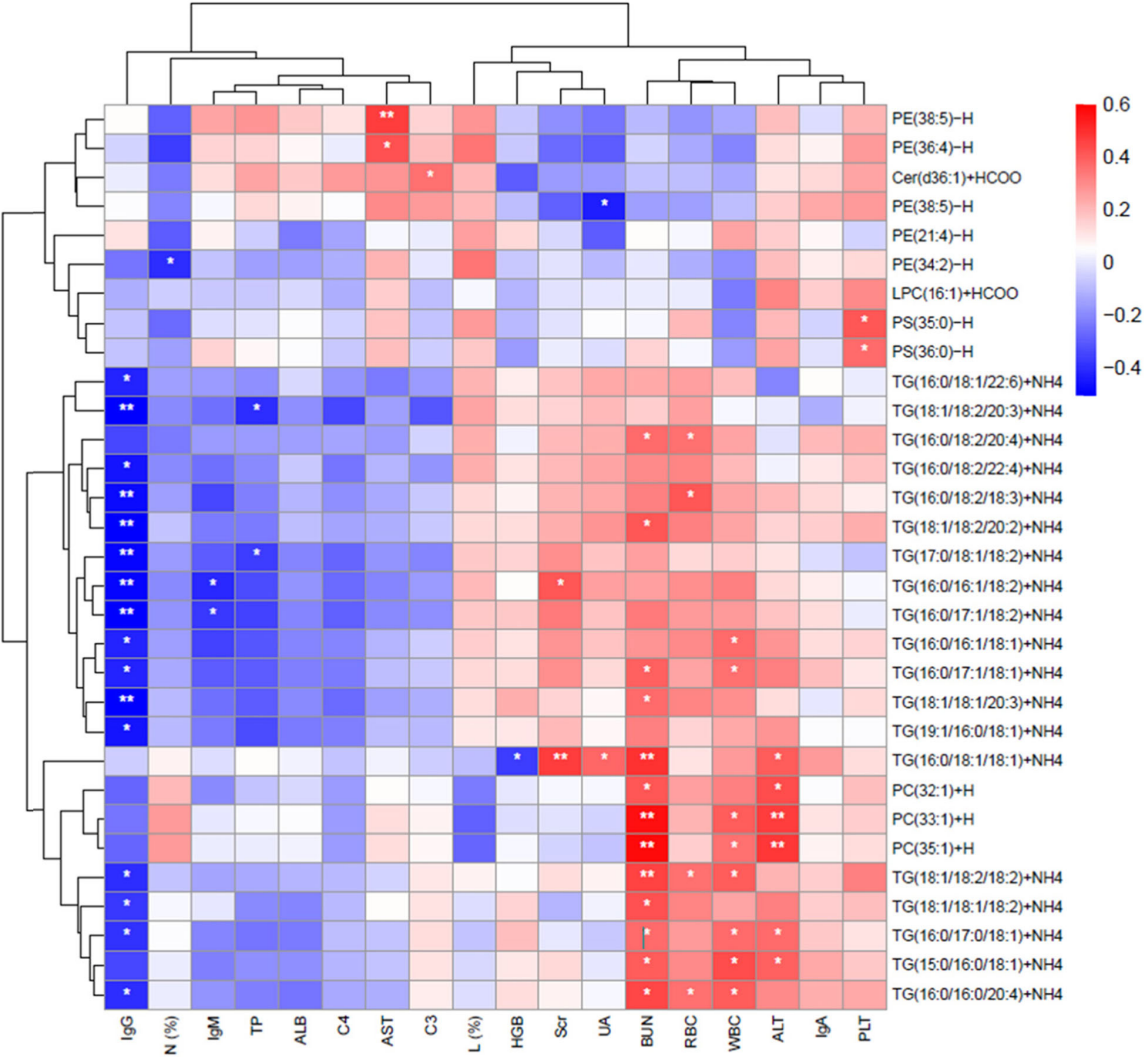
The heat plot for the 31 lipid ions and other lab examinations. Negative correlation colored blue and positive correlation colored red. * means *p* < 0.05, and ** means *p* < 0.01

Thirty One identified lipidions showed significant difference between HCs and IgAVs, and the detailed information of them is provided in Table 2. The heat plot for these differential lipids is shown in Fig. 2. As it can be seen in the heat plot, the plasma lipid levels of IgAV patients are increased over all. All these 31 lipid ions can be divided into 6 classes, including TG, PE, PC, Phosphatidylserine (PS), Cer, and lyso-phosphatidylcholine (LPC). Most of these lipidions are TGs. The correlation between 31 lipid ions and laboratary examinations were showed in Fig. 3.

**Table 2:** Identified differential lipidions between HCs and IgAVs

Lipidions	Class	Fatty Acid	VIP	***p*** value	Fold.change	Log1.5 foldchange
TG(16:0/18:2/18:3) + NH4	TG	(16:0/18:2/18:3)	1.84835	0.02482772	2.103717	1.834204
TG(15:0/16:0/18:1) + NH4	TG	(15:0/16:0/18:1)	1.37775	0.04404364	1.923537	1.613371
TG(16:0/16:0/20:4) + NH4	TG	(16:0/16:0/20:4)	1.72933	0.00999953	1.909782	1.595671
TG(16:0/17:1/18:2) + NH4	TG	(16:0/17:1/18:2)	1.62306	0.00722187	1.80384	1.454916
PC(32:1) + H	PC	(32:1)	2.56503	0.00066097	1.796607	1.445007
PS(35:0)-H	PS	(35:0)	3.2507	0.00036811	1.780378	1.422627
TG(16:0/17:1/18:1) + NH4	TG	(16:0/17:1/18:1)	2.23389	0.00898277	1.768255	1.405776
TG(18:1/18:2/18:2) + NH4	TG	(18:1/18:2/18:2)	3.78748	0.00043832	1.757964	1.391381
TG(16:0/18:2/20:4) + NH4	TG	(16:0/18:2/20:4)	2.6048	0.00040886	1.748873	1.378594
PC(33:1) + H	PC	(33:1)	1.25795	0.0002234	1.7368	1.361509
TG(16:0/17:0/18:1) + NH4	TG	(16:0/17:0/18:1)	1.32552	0.02861302	1.736614	1.361245
TG(18:1/18:1/18:2) + NH4	TG	(18:1/18:1/18:2)	1.19282	0.00397713	1.713335	1.327961
PS(36:0)-H	PS	(18:0/18:0)	1.54875	0.00017949	1.668692	1.262846
Cer(d36:1) + HCOO	Cer	(d18:1/18:0)	1.36673	0.00092262	1.654524	1.241817
TG(16:0/16:1/18:1) + NH4	TG	(16:0/16:1/18:1)	5.6857	0.01808469	1.620904	1.191185
TG(18:1/18:2/20:2) + NH4	TG	(18:1/18:2/20:2)	1.9634	9.50E-05	1.616611	1.184644
TG(16:0/18:1/18:1) + NH4	TG	(16:0/18:1/18:1)	1.60668	0.02201249	1.592181	1.14709
TG(18:1/18:1/20:3) + NH4	TG	(18:1/18:1/20:3)	1.8557	0.00061773	1.583948	1.134303
TG(19:1/16:0/18:1) + NH4	TG	(19:1/16:0/18:1)	1.50919	0.01516599	1.577544	1.124312
TG(16:0/18:1/22:6) + NH4	TG	(16:0/18:1/22:6)	1.88274	0.00046975	1.557257	1.09239
PE(36:4)-H	PE	(16:0/20:4)	1.77295	0.00246647	1.55075	1.082063
TG(16:0/16:1/18:2) + NH4	TG	(16:0/16:1/18:2)	4.49804	0.01947916	1.547024	1.07613
PE(38:5)-H	PE	(18:0/20:5)	1.02753	0.01636181	1.541867	1.067895
PC(35:1) + H	PC	(35:1)	1.177	0.00014923	1.529081	1.047357
TG(17:0/18:1/18:2) + NH4	TG	(17:0/18:1/18:2)	1.41525	0.01595431	1.509847	1.016138
LPC(16:1) + HCOO	LPC	(16:1)	1.37718	3.72E-05	1.508243	1.013516
PE(38:5)-H	PE	(18:1/20:4)	1.09762	0.01334339	1.506316	1.010363
TG(16:0/18:2/22:4) + NH4	TG	(16:0/18:2/22:4)	3.00908	0.00181107	1.505923	1.009719
PE(34:2)-H	PE	(16:0/18:2)	1.60924	0.01677844	1.505824	1.009557
TG(18:1/18:2/20:3) + NH4	TG	(18:1/18:2/20:3)	1.01849	0.00323496	1.505755	1.009444
PE(21:4)-H	PE	(10:0/11:4)	1.41852	0.03854884	0.601425	−1.254

**Fig. 4:**
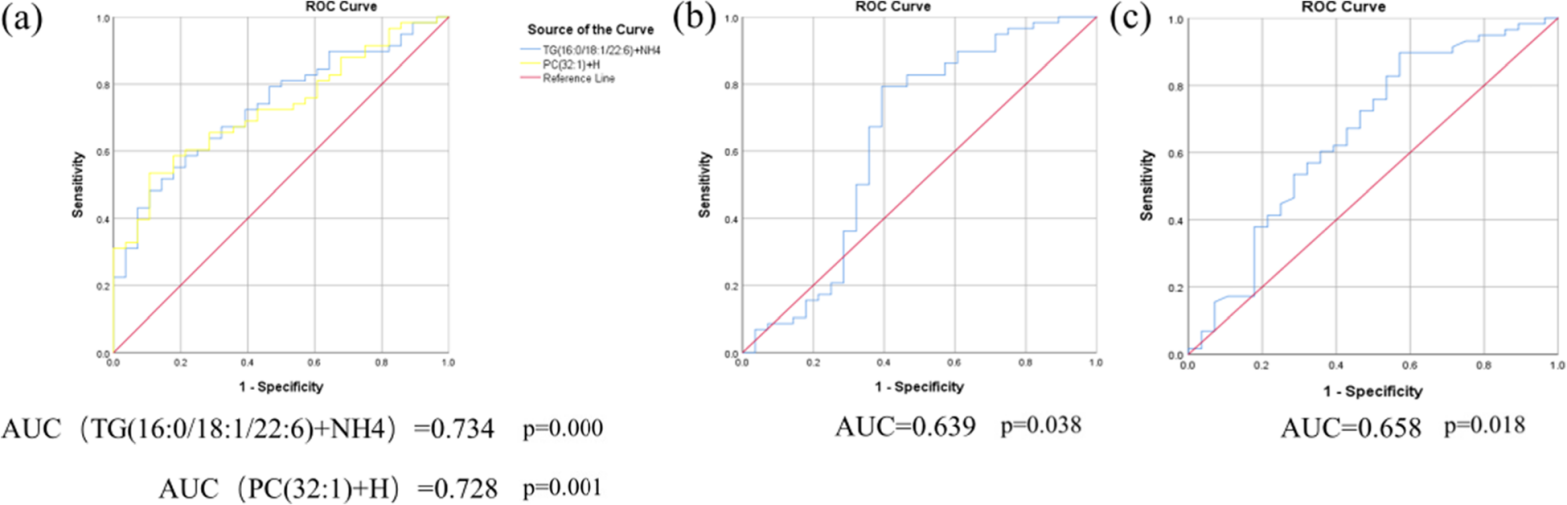
The ROC Curve of 2 increased lipidions(a), 1 decreased lipidions(b) and BMI(c) for IgAV

To further evaluate the lipid biomarkers of IgAV, a stepwise binary logisticregression analysis regressed all these 31 lipid ions and BMI and showed the model of TG (16:0/18:1/22:6) +NH4, PC (32:1) +H, BMI, and PE (21:4)-H was significant with the correct class of 83.7%. A decreased BMI was linked with an increased risk of IgAV (OR=0.68, 95%CI: 0.53~0.88). Among these three lipid ions, PE (21:4)-H were decreased (FC<1) in IgAVs. ROC analysis was performed and corresponding AUC was calculated to evaluate the diagnostic accuracy of each of the 3 plasma lipid ions and BMI for diagnosing IgAV (Fig. 4). TG (16:0/18:1/22:6) +NH4, and PC (32:1) +H were significantly increased, with AUC greater than 0.7, distinguishing IgAV patients from HCs. Then we determined cut-points for TG (16:0/18:1/22:6) +NH4 (888875609.05), PC (32:1) +H (905307459.90), BMI (19.18), and PE (21:4)-H (32236196.59) (Table 3). Children with the quantity of TG(16:0/18:1/22:6)+NH4 over 888875609.05 were 4.974 times more likely with IgAV (OR=4.974, 95%CI: 1.027~24.092); Children with the quantity of PC(32:1)+H over 905307459.90 were 9.804 times more likely with IgAV (OR=9.804, 95%CI: 1.964~48.942); children with the quantity of PE(21:4)-H less than 32236196.59 were 6.897 times more likely with IgAV (OR=6.897,95%CI: 1.920~24.774); children with BMI less than 19.18 were 5.629 times more likely with IgAV (OR=5.629, 95%CI: 1.4445~21.937).

**Table 3:** TG(16:0/18:1/22:6) + NH4, PC(32:1) + H, PE (21:4)-H, and BMI as predictors of IgAV

Parameter	B	*Std. Erro*r	Wald Chi-Square	*p* Value	Odds Ratio	95%CI	
						Lower	Upper
TG(16:0/18:1/22:6) + NH4	1.604	0.805	3.971	0.046	4.974	1.027	24.092
PC(32:1) + H	2.283	0.820	7.744	0.005	9.804	1.964	48.942
PE(21:4)-H	1.931	0.652	8.761	0.003	6.897	1.920	24.774
BMI	1.728	0.694	6.199	0.0132	5.629	1.4445	21.937

### Lipidomics analysis of plasma obtained from IgAV-N subjects and IgAV-C

**Fig. 5:**
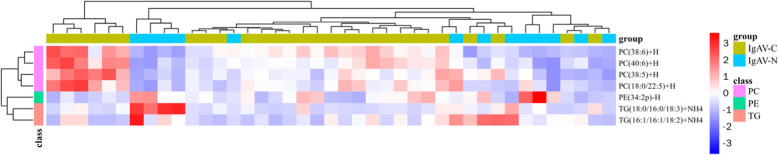
The heat plot for the differential lipids in IgAV-N vs IgAV-C. Decreased lipidions colored blue and increased lipidions colored red. PCs were decreased in IgAVN, while PEs and TGs were increased

**Fig. 6:**
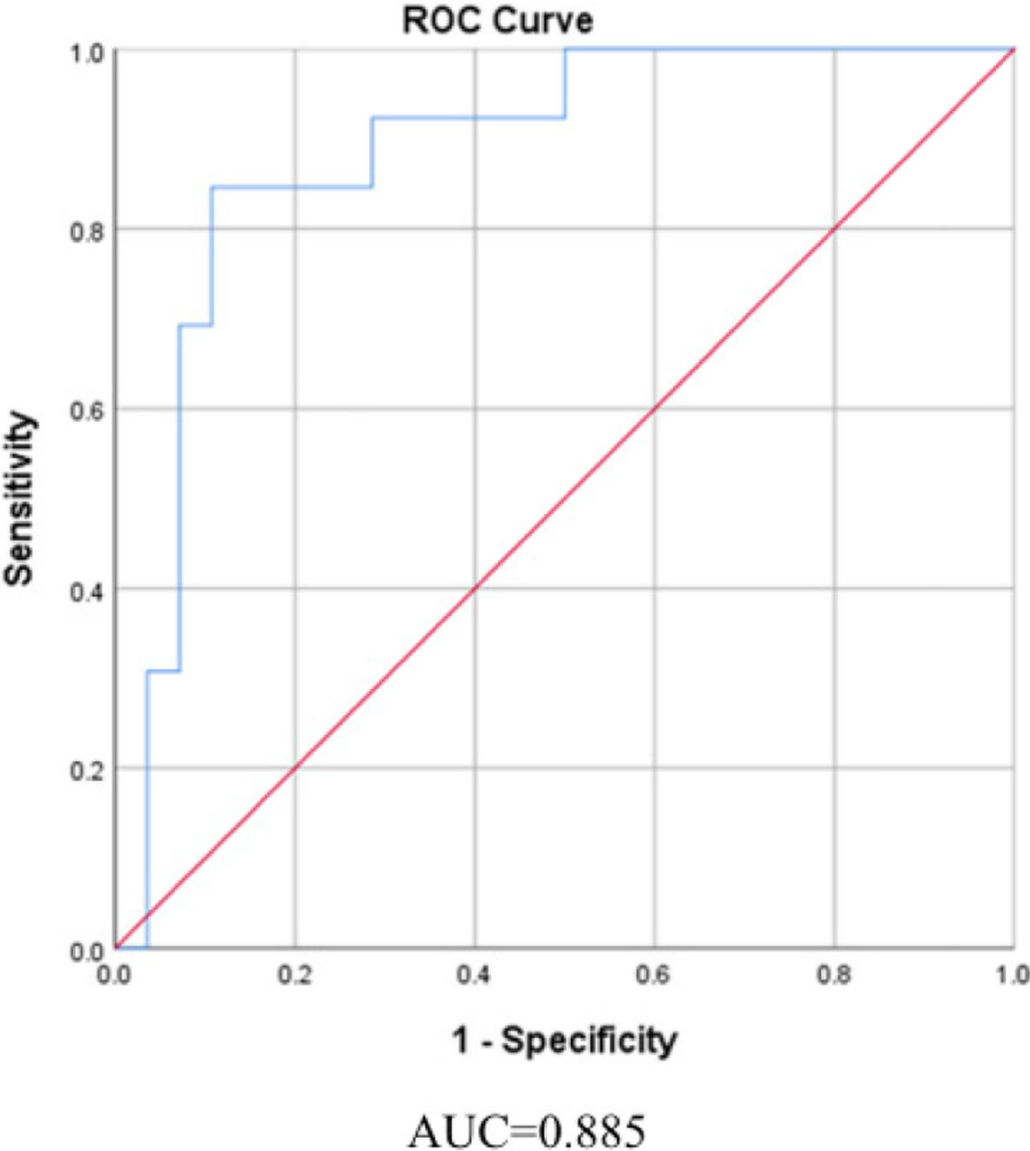
The ROC Curve of PC (38:6) + H for IgAVN

Seven kinds of lipidions were found to be significantly different (p<0.05, VIP>1, FC>1.5) (Table 4), and a heatmap was performed in Fig. 5. PC (38:6) +H, was significantly different (p<0.01) with correct class of 85.4% (Table S3). PC (38:6) +H was decreased in IgAVN patients with an AUC of 0.885 to discriminate IgAV-N from IgAV-C (Fig. 6). We identified the cut-point of PC (38:6) (4469726623) for IgAVN on the basis of the results from the ROC curve. Based on the logistic model with PC (38:6), children with the quantity of PC (38:6) less than 4469726623 were 45.833 times more likely with IgAVN (OR=45.833, 95% CI: 6.689~341.070).

**Table 4:** Identified differential lipid ions between IgAVc and IgAVN

LipidIons	Class	Fatty Acid	***p*** value	VIP	Fold change	log1.5 fold change
TG(18:0/16:0/18:3) + NH4	TG	(18:0/16:0/18:3)	0.0013	2.57394	3.28158	2.93077
TG(16:1/16:1/18:2) + NH4	TG	(16:1/16:1/18:2)	0.014209	1.49978	2.01381	1.726483
PE(34:2p)-H	PE	(34:2p)	0.016049	1.27288	1.52816	1.045871
PC(38:5) + H	PC	(18:1/20:4)	0.002901	2.87729	0.662728	−1.01461
PC(38:6) + H	PC	(16:0/22:6)	5.55E-05	3.21673	0.649622	−1.06388
PC(18:0/22:5) + H	PC	(18:0/22:5)	0.008981	1.27596	0.635838	− 1.11677
PC(40:6) + H	PC	(40:6)	0.000125	2.6263	0.608873	−1.22365

### Lipidomics analysis between the plasma of IgAV1 and IgAV0

A lipidions contrast was made between IgAV0 and IgAV1 to evaluate the lipidions changes during the treat phrase with glucocorticoids. 17 lipidions were identified to be significantly different (*p* < 0.05, VIP>1, FC>1.5) (Table 5). TG (16:0/18:1/22:6) +NH4, TG (16:0/16:1/18:2) +NH4, and TG (16:0/18:2/18:3) +NH4 were significantly different in both IgAV1vs IgAV0 and IgAVs vs HCs (Table 6) and remained elevated after treatment.

**Table 5:** Identified differential lipid ions between IgAV0and IgAV1

LipidIons	Class	Fatty Acid	***p*** value	VIP	Fold change	log1.5 fold change
SM(d18:1/24:1) + H	SM	(d18:1/24:1)	0.00484	5.8611	0.734012	−7.63E-01
TG(18:3/18:2/18:2) + NH4	TG	(18:3/18:2/18:2)	0.01953	2.73572	4.22322	3.55E+ 00
TG(16:1/18:2/18:3) + NH4	TG	(16:1/18:2/18:3)	0.0158979	1.29336	3.19441	2.86E+ 00
TG(16:1/16:1/18:2) + NH4	TG	(16:1/16:1/18:2)	0.007937	1.16421	2.82852	2.56E+ 00
TG(16:0/18:1/22:6) + NH4	TG	(16:0/18:1/22:6)	0.0090351	1.37791	2.71566	2.46E+ 00
TG(18:3/18:2/18:2) + Na	TG	(18:3/18:2/18:2)	0.00191	1.06146	2.55724	2.32E+ 00
TG(18:1/18:2/18:3) + NH4	TG	(18:1/18:2/18:3)	0.0076853	3.27982	2.09544	1.82E+ 00
PC(32:2) + H	PC	(14:0/18:2)	0.0075443	1.44772	1.99826	1.71E+ 00
TG(18:1/18:1/18:3) + NH4	TG	(18:1/18:1/18:3)	0.0037147	2.4811	1.82271	1.48E+ 00
PC(36:4) + H	PC	(18:2/18:2)	0.0265343	1.97895	1.79346	1.44E+ 00
TG(16:0/18:2/18:3) + NH4	TG	(16:0/18:2/18:3)	0.0271601	1.92436	1.75106	1.38E+ 00
TG(16:0/16:1/18:2) + NH4	TG	(16:0/16:1/18:2)	0.0379257	1.65399	1.66646	1.26E+ 00
TG(16:0/18:1/22:5) + NH4	TG	(16:0/18:1/22:5)	0.0135847	1.84413	1.58691	1.14E+ 00
PC(34:2e) + H	PC	(34:2e)	0.0298178	2.96902	1.57944	1.13E+ 00
LPC(20:5) + H	LPC	(20:5)	0.0232202	1.05066	1.51736	1.03E+ 00
TG(18:1/18:2/22:5) + NH4	TG	(18:1/18:2/22:5)	0.0358588	1.93212	0.592921	−1.29E+ 00
SM(d42:1) + H	SM	(d42:1)	0.0263021	1.59175	0.468622	−1.87E+ 00

**Table 6:** 3 lipidions significantly different in both IgAVs vs HCs and IgAV1 vs IgAV0

Lipidions	Fold Change	
	IgAVs vs HCs	IgAV1 vs IgAV0
TG(16:0/18:1/22:6) + NH4	1.557256907	2.71566
TG(16:0/16:1/18:2) + NH4	1.54702425	1.66646
TG(16:0/18:2/18:3) + NH4	2.103717277	1.75106

## Discussion

The current study was carried out to investigate the lipidomic profile for the patients with IgAV in order to established and evaluate them as novel biomarkers.

Among IgAV patients and healthy controls, there were no differences in age or sex, whereas BMI was significantly increased in IgAV patients. GM3 was significantly associated (*p* = 0.000454) with IgAV, though the FC was small. 31 lipidions were finded to be significantly changed in IgAV patients and all of them are divided into 6 classes, including TG, PE, PC, PS, Cer and LPC. Most of these 31 lipidions were increased in IgAV patients, apart of PE (21:4)-H.

Among IgAVN patients and IgAV patients without nephritis, no difference in basic characteristics was observed. And there are two different change trends of lipidions in IgAVN patients and IgAV patients without nephritis that in IgAVN, PEs and TGs continued growing while PCs declined.

Most of these significant lipidions in IgAV belong to TG which plays an important role in cardiovascular diseases, which are also common seen in IgAVs [28]. High TG concentrations are associated with decreased high-density lipoprotein (HDL) cholesterol (HDL-C) levels, elevated levels of non–HDL-C and raised remnants rich in cholesterol, that are risks of cardiovascular disease [29, 30]. Also, TG is are correlated with the severity of insulin resistance, which has been showed to have an adverse impact on cardiovascular and renal systems [31]. Moreover, Metabolic pathways of TG are not independent from the effects of the presence of structural lipids such as glycerophospholipids or sphingolipids, which may interact with TG metabolism at multiple levels [32]. PC is one of GPs and it has been reported to prevent TG accumulation and the coalescence of LDs (lipid droplets), which are cellular storage organelles for TG [33]. Meanwhile, LDs can synthesize PC locally with 2 PC synthesizing enzymes, lysophosphatidylcholine acyltransferase 1 and lysop hosphatidylcholine acyltransferase 2, localized on the surface of LDs [34]. Besides, Cer was involved in the process that TG influences the severity of insulin resistance [35–37]. In our research, TG are positive related to BUN and WBC. BUN is generally regarded as a significant serum marker in estimating renal function which is important in IgAV. WBC are involved in immunity which related to the pathogenesis of IgAV [38].

In addition, LPC is called as ‘the grease for cardiovascular disease’, and it can activate platelets as well as form inflammatory platelet-monocyte aggregates interaction with G2AR on platelets [39, 40]. In IgAV children, platelet is in a high level [41]. Platelets not only exacerbate cardiovascular diseases, but also aggravate inflammation. Activated platelets can induce the release of inflammatory cytokines (including TNF-α and IL-6) and the presentation of surface molecules (including P-selectin, E-selectin) to recruit neutrophil and release NETosis [41, 42]. Meanwhile, platelet can release C3 and GM-CSF, which also contribute to IgAV.

Plasmalogen PC and plasmalogen PE were reported to be associated with oxidative stresses which are important in IgAV pathogenesis [43]. In our result PE(36:4)-H, PE (38:5)-H, PE (38:5)-H PE (34:2)-H and PCs rose, while PE (21:4)-H declined. In addition, the diversity of PE and PC species containing polyunsaturated fatty acyls (acids) (PUFAs) contributes to the production of lipid mediators [44, 45]. The decreased PE (21:4)-H may be hydrolyzed to release free arachidonic acid, to release varieties of eicosanoids such as prostaglandins and leukotrienes, causing inflammation and damage of vascular endothelial cells in IgAV. These increased phospholipids may rise to compensate for the reduced PE (21:4)-H, as well as be the reservoir for producing anti-inflammatory mediators such as resolvins and protectins [25].

PS were reported to be associated with mitochondrial function, which plays an important role in cell death such as apoptosis [46]. PS exposed on the cell surface is an apoptotic ‘eat me’ signal which can be recognized by the PS receptor, or a secreted PS-binding molecules, which bind PS on apoptotic cells and a membrane protein on the macrophage [46]. Besides, in our study, PS are found to be related to platelets count.

Cer belong to SP. SP plays an important role in cell cycle arrest, apoptosis, and cell senescence [47, 48]. Cer can upregulate cytokine expression and cell apoptosis through inducing the activity of caspase3 and caspase8 [49, 50].

Though the change of GM3 was little, GM3 may be involved in the pathogenesis of IgAV. GM3 was found to inhibit cell growth by decreasing cell adhesion and epidermal growth factor-dependent phosphorylation of epidermal growth factor receptor [51]. GM3 were also report to increase IL-17 proliferation and secretion from Th17 cells so as to accelerate the pathogenesis and progression of RA and mouse CIA [52]. In human B cell, GM3 can inhibit IgG subclasses and IgM production, but not IgA1 and IgA2 [53].

Above all, lipid metabolism can participate in the pathogenesis of IgAV, through cardiovascular disease, insulin resistance, cell apoptosis, and inflammation.

The multiple logistic regression analysis was used to assess associations between lipidions and IgAV. Based on the lipidions quantitated by LC/MS, we constructed ROC curves to select the optimal cut-point for TG(16:0/18:1/22:6) + NH4 (888,875,609.05), PC(32:1) + H (905,307,459.90), and PE (21:4)-H (32,236,196.59). Then, we started multiple logistic regression to prove that the increase in TG(16:0/18:1/22:6) + NH4 and PC(32:1) + H, as well as the decrease of PE (21:4)-H can raise risk of IgAV. Besides, in our study BMI also decreased in IgAVs, that is not normal in cardiovascular diseases [54]. This may because the average BMI of both IgAVs and HCs fallen within the normal BMI range.

Next, we compared IgAV patients with and without nephritis. PCs were decreased in IgAVN while PEs and TGs were increased. In our study, PC (38:6) + H was found to be the most prominent biomarker in IgAVN, with a significant decrease and an AUC of 0.885. As an anti-inflammatory and antioxidant agent, PC ameliorated oxidative stress in kidney via reducing proinflammatory cytokines TNF-α and IL-6 [55]. PC also decreased tubular degeneration and hypertrophy of glomeruli by enhancing antioxidant enzyme activity [56]. Meanwhile, DHA-enriched PC can attenuate nephrotoxicity through inactivating mitogen-activated protein kinase (MAPK) signaling pathways, including upregulation of Bcl-2 and downregulation of caspase-9, caspase-3, cytochrome-c, p38, and JNK. On the contract, PC loss may lead to renal damage. In animals, PC mainly obtained from diet and the methylation of PE by the enzyme PE *N*-methyltransferase (PEMT) [57]. The increased PE and decreased PC imply that the methylation of PE may be disturbed in IgAVN.

Finally, comparison before treatment and after treatment with glucocorticoids was made. In our research, TG was increased in IgAV and continued to rise after threaten. That could be because of the effects of glucocorticoids [58].

This study reveals the lipid alterations in IgAV and IgAVN. However, the mechanisms of lipid alterations can only be speculated upon at present. Further studies are required to explore the functions of theses lipids in physiological and pathological processes through cell and animal experiments.

## Supplementary Information

**Additional file 1.**

## Abbreviations

IgAV: IgA vasculitis
IgAVN: IgA vasculitis nephritis
FA: Fatty acyls
GL: Glycerolipids
GP: Glycerophospholipids
SP: Sphingolipids
ST: Sterol lipids
PR: Prenol lipids
SL: Saccharolipids
PK: Polyketides
MG: Monoacylglycerols
DG: Diacylglycerols
TG: Triacylglycerols
PG: Phosphatidylglycerol
PC: Phosphatidylcholine
PE: Phosphatidylethanolamine
Cer: Ceramide
PON1: Paraoxonase1
HDL: High density lipoprotein
HDL-C: High-density lipoprotein cholesterol
LDs: Lipid droplets
LC-MS/MS: Liquid Chromatography with tandem mass spectrometry
ESI: Electrospray ionization
VIP: Variable importance of the projection
BUN: Blood urea nitrogen
Scr: Serum creatinine
UA: Uric acid
WBC: White blood cell
RBC: Red blood cell
HGB: Hemoglobin concentration
PLT: Platelets count
N (%): Neutrophil count
L (%): Lymphocyte count
C3: Complement component 3
C4: Complement component 4
ALT: Alanine aminotransferase
AST: Aspartate aminotransferase
ALB: Albumin
TP: Total protein
