# Supplementary material for: Lipid metabolism contribute to the pathogenesis of IgA Vasculitis
Source: Diagn Pathol. 2022 Feb 11;17:28. doi: 10.1186/s13000-021-01185-1 (PMC8840790; doi:10.1186/s13000-021-01185-1)
Supplement: Supplementary file 1 — Additional file 1. [file 13000_2021_1185_MOESM1_ESM.docx]

# Supplementary material


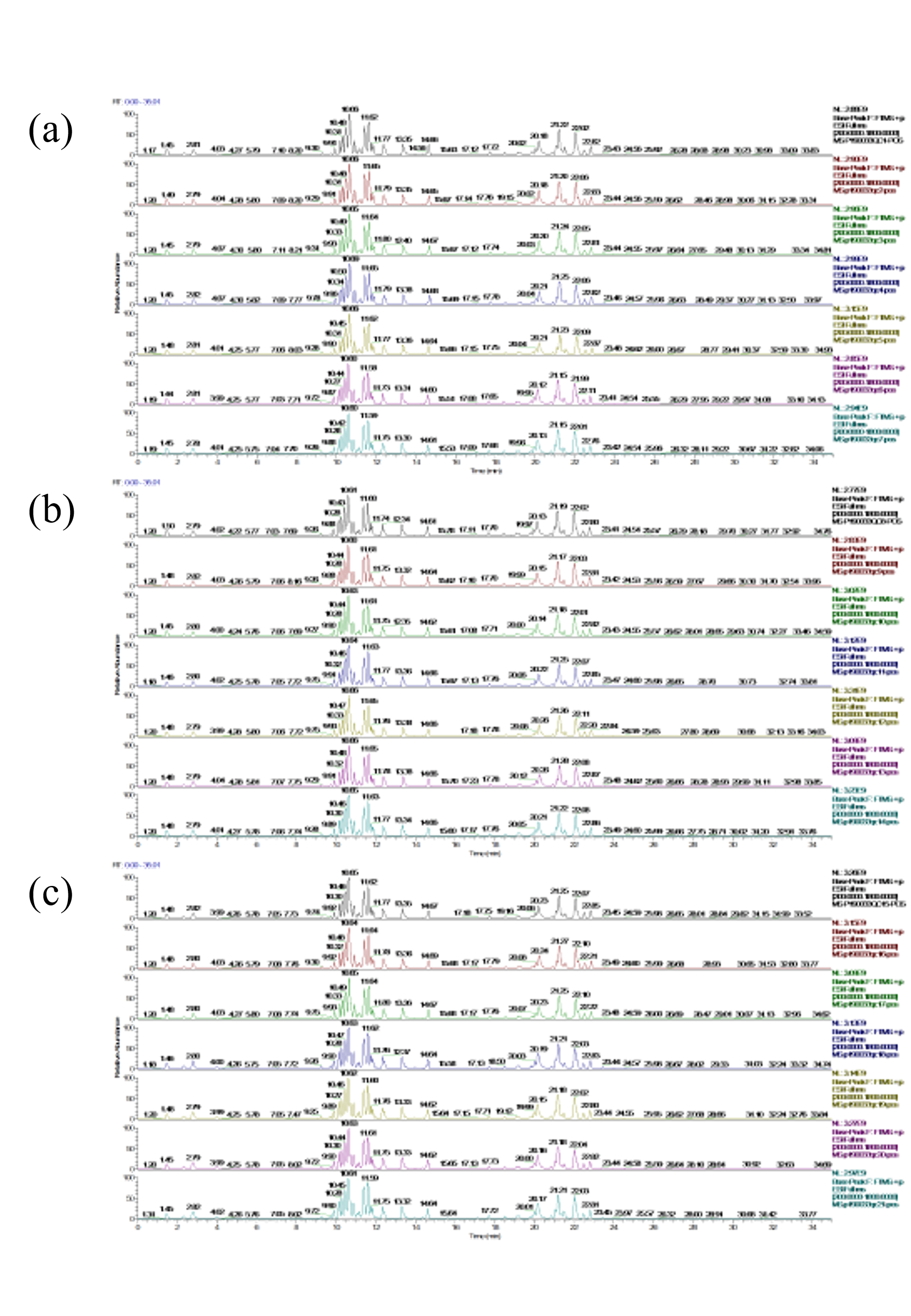


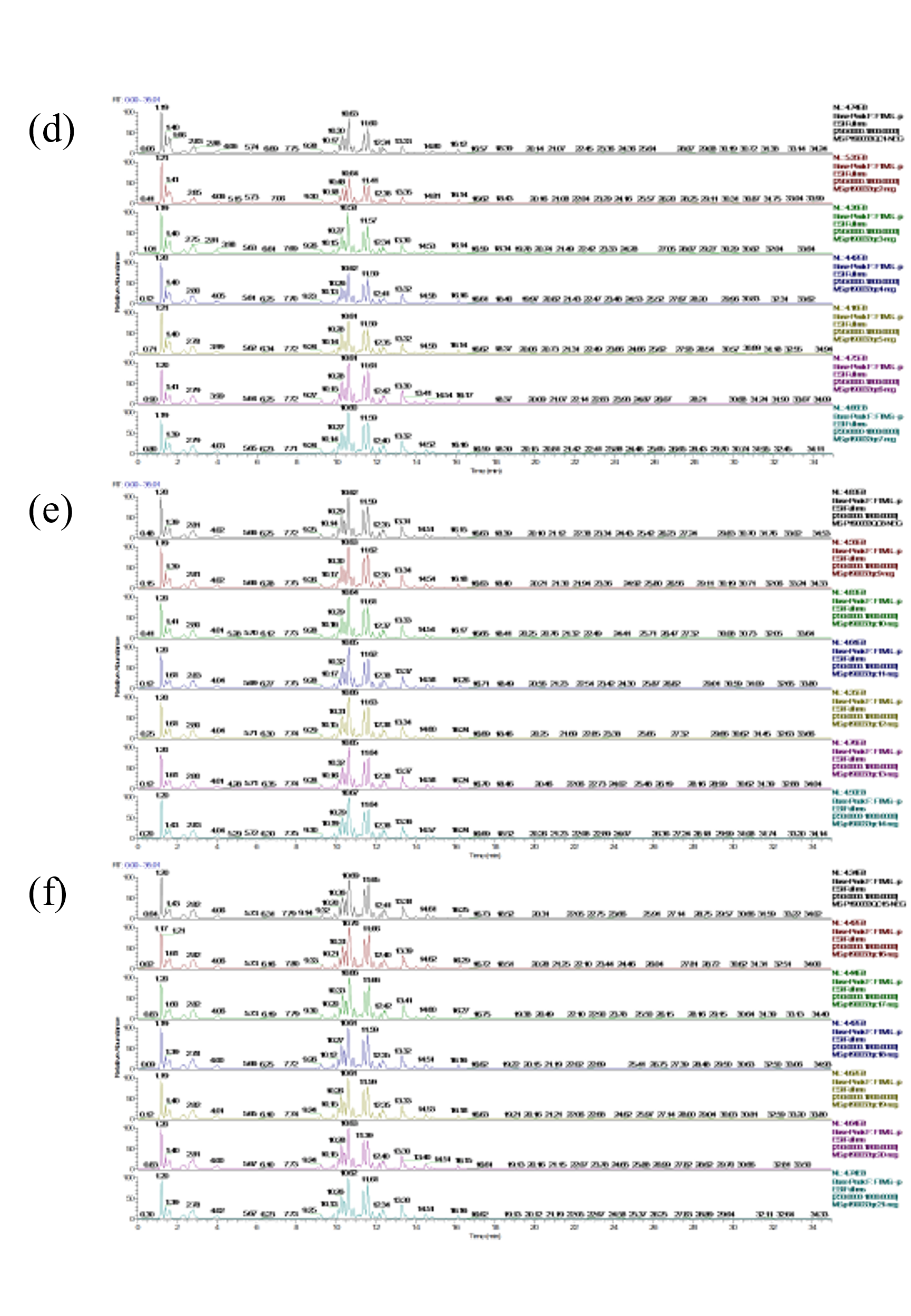


**Figure S1** the basic peak chromatogram (BPC) of QC samples in positive ion mode.

**
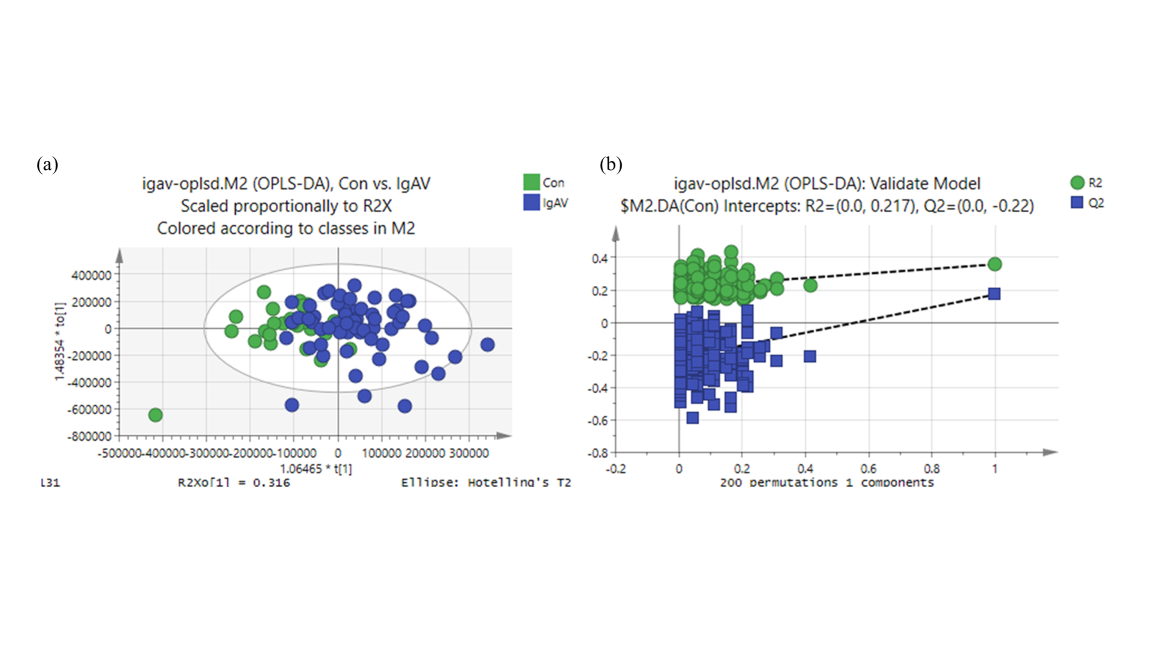
**

**Figure S2** OPLS-DA score plot of samples(a) and permutation test of samples(b).

**Table S1.** Stepwise binary logistic-regression models for selecting potential lipid biomarkers for IgAV

| **Model** | **Parameter** | **B** | ***Std. Erro*r** | **Wald Chi-Square** | ***p* Value** | **Correct Class %** |
| --- | --- | --- | --- | --- | --- | --- |
| Model 1 | TG(16:0/18:1/22:6)+NH4 | 0.000 | 0.000 | 9.760 | 0.002 | 68.6% |
| Model 2 | PC(32:1)+H | 0.000 | 0.000 | 6.248 | 0.012 | 74.4% |
|  | TG(16:0/18:1/22:6)+NH4 | 0.000 | 0.000 | 6.962 | 0.008 |  |
| Model 3 | PC(32:1)+H | 0.000 | 0.000 | 7.268 | 0.007 | 79.1% |
|  | TG(16:0/18:1/22:6)+NH4 | 0.000 | 0.000 | 8.253 | 0.004 |  |
|  | bmi | -0.228 | 0.084 | 7.319 | 0.007 |  |
| Step 4 | PC(32:1)+H | 0.000 | 0.000 | 6.912 | 0.009 | 83.7% |
|  | TG(16:0/18:1/22:6)+NH4 | 0.000 | 0.000 | 7.914 | 0.005 |  |
|  | PE(21:4)-H | 0.000 | 0.000 | 6.784 | 0.009 |  |
|  | bmi | -0.270 | 0.102 | 6.947 | 0.008 |  |
| Model 5 | PC(32:1)+H | 0.000 | 0.000 | 9.041 | 0.003 | 83.7% |
|  | TG(18:1/18:1/18:2)+NH4 | 0.000 | 0.000 | 5.631 | 0.018 |  |
|  | TG(16:0/18:1/22:6)+NH4 | 0.000 | 0.000 | 10.484 | 0.001 |  |
|  | PE(21:4)-H | 0.000 | 0.000 | 6.377 | 0.012 |  |
|  | BMI | -0.331 | 0.119 | 7.679 | 0.006 |  |
| Model 6 | PC(32:1)+H | 0.000 | 0.000 | 7.554 | 0.006 | 83.7% |
|  | TG(18:1/18:1/18:2)+NH4 | 0.000 | 0.000 | 4.609 | 0.032 |  |
|  | TG(16:0/18:1/22:6)+NH4 | 0.000 | 0.000 | 9.633 | 0.002 |  |
|  | LPC(16:1)+HCOO | 0.000 | 0.000 | 4.427 | 0.035 |  |
|  | PE(21:4)-H | 0.000 | 0.000 | 6.840 | 0.009 |  |
|  | BMI | -0.384 | 0.131 | 8.590 | 0.003 |  |

**Table S2.** Stepwise binary logistic-regression models for selecting potential lipid biomarkers for IgAVN

| **Model** | **Parameter** | **B** | ***Std. Erro*r** | **Wald Chi-Square** | ***p* Value** | **Correct Class %** |
| --- | --- | --- | --- | --- | --- | --- |
| Model 1 | PC(38:6)+H | 0 | 0 | 9.846879115 | 0.001701201 | 85.37% |
